# Why Can Modulation of α6-Containing GABA_A_ Receptors Reduce the Symptoms of Multiple Neuropsychiatric Disorders?

**DOI:** 10.33696/Pharmacol.6.047

**Published:** 2024-01

**Authors:** Werner Sieghart

**Affiliations:** 1 Center for Brain Research, Department of Molecular Neurosciences, Medical University Vienna, Spitalgasse 4, A-1090 Vienna, Austria

**Keywords:** GABA_A_ receptors, α6-containing GABA_A_ receptors, Positive modulators selective for α6-containing GABA_A_ receptors, Cerebellar granule cells, Cerebellar function in the brain, Neuropsychiatric disorders, Trigeminal-related pain

## Abstract

α6-containing GABA_A_ receptors (α6GABA_A_Rs) are strongly expressed in cerebellar granule cells, where they mediate a correctly timed and precise coordination of all muscle groups that execute behavior and protect the brain from information overflow. Recently, it was demonstrated that positive modulators with a high selectivity for α6GABA_A_Rs (α6-modulators) can reduce the symptoms of multiple neuropsychiatric disorders in respective animal models to an extent comparable with established clinical therapeutics. Here, these incredible findings are discussed and explained. So far, the beneficial actions of α6-modulators and their lack of side effects have only been demonstrated in animal models of the respective disorders. Preclinical studies have demonstrated their suitability for further drug development. Future human studies have to investigate their safety and possible side effects, and to clarify to which extent individual symptoms of the respective disorders can be reduced by α6-modulators in patients during acute and chronic dosing. Due to their broad therapeutic potential, α6-modulators might become a valuable new treatment option for multiple neuropsychiatric disorders.

## Introduction

### GABA_A_ receptors

GABA_A_ receptors (GABA_A_Rs) are the major inhibitory neurotransmitter receptors in the brain and the site of action of a variety of clinically important drugs, such as benzodiazepines, barbiturates, anesthetics, neuroactive steroids, and convulsants [1]. They are chloride ion channels that can be opened by GABA and are composed of five from a total of 19 subunits, that can form a multiplicity of GABA_A_R subtypes. The majority of these receptor subtypes is composed of 2α, 2β, and one γ or δ subunit [2]. Benzodiazepines are positive modulators of GABA_A_Rs that exert their anticonvulsant, muscle relaxant, sedative hypnotic, and anxiolytic activity by enhancing the action of GABA at the benzodiazepine-sensitive α1βγ2, α2βγ2, α3βγ2, and α5βγ2 receptors. These receptors differentially contribute to the activities of benzodiazepines. α1βγ2 receptors predominantly contribute to the sedative-hypnotic, anterograde amnesia, and anticonvulsant properties. α2βγ2 receptors mediate anxiolytic, and together with α3βγ2 receptors, the analgetic activity of benzodiazepines. α2βγ2, α3βγ2, and α5βγ2 GABA_A_Rs contribute to the myorelaxant activity, and α5βγ2 receptors can modulate learning and memory [3].

To generate drugs with more selective actions, a variety of benzodiazepine binding site ligands from different structural classes have been developed that are able to differentially interact with benzodiazepine-sensitive GABA_A_R subtypes. However, the currently available compounds are not sufficiently selective and still modulate other receptor subtypes at comparable concentrations. Although their differential efficacy at distinct receptor subtypes reduced side effects in behavioral experiments in rodents, discrepant *in vivo* effects of some of these compounds in rodents and humans raised doubts on the applicability of the concept of receptor subtype-selectivity as a guide for the development of clinically useful drugs [4,5].

### α6-containing GABA_A_ receptors

GABA_A_R subtypes composed of α4βγ2, α6βγ2, or αβδ subunits cannot be modulated by benzodiazepines and are thus benzodiazepine-insensitive. Especially GABA_A_Rs composed of α6βγ2 and α6βδ subunits (α6GABA_A_Rs) are also much less abundant and for many years it seemed that they are expressed in cerebellar granule cells, only. Their overall importance seemed thus to be low, and they were not much investigated. In the meantime, however, evidence accumulated that these receptors are also widely expressed in other parts of the brain as well as in peripheral tissues, but at a low abundance [6]. In addition, the identification of some pyrazoloquinolinones as the first highly selective positive modulators of α6GABA_A_Rs (α6-modulators), that are not able to modulate the much more abundant benzodiazepine-sensitive GABA_A_Rs up to μM concentrations [7], changed the picture. Since that, several studies indicated that these α6-modulators in animal models can reduce the symptoms of tic disorders, such as Tourette Syndrome [8,9], schizophrenia [10], and essential tremor [11], to an extent comparable with standard therapeutics used to treat these disorders. In animal models of schizophrenia these compounds not only reduced positive symptoms but also negative symptoms and cognitive impairment [10]. All these actions seemed to be mediated via α6GABA_A_Rs in the cerebellum. These incredible findings raise the question, how all that can be explained and how these apparently unimportant GABA_A_Rs are able to do that!

In this narrative review, the structure, function, and organization of the cerebellum and its integration in neuronal circuits of the whole brain is discussed, and the function and importance of α6GABA_A_Rs in the cerebellum and in other parts of the nervous system is explained. Lesions in the cerebellum of patients accompanied by symptoms of neuropsychiatric disorders suggest a cerebellar contribution to these disorders. Changes in the structure, function, and connectivity of the cerebellum identified in neuropsychiatric patients support this conclusion. Human and animal studies demonstrating an association between changes of α6GABA_A_R expression or function, and the occurrence of neuropsychiatric symptoms are summarized. Amelioration of such symptoms by α6-modulators in animal models of neuropsychiatric disorders is discussed. The review concludes with the current status of development of α6-modulators and their future prospects.

## Structure and Function of the Cerebellum

The cerebellar cortex consists of a folded sheet of microcircuitry reiterated on a vast scale. After receiving specific sensory and motoric information, this circuitry predicts time intervals for motor activity required for controlling movement of the respective body parts. The same circuitry in different areas of the cerebellum probably computes similar operations linked to different parts of the brain [6]. This microcircuitry has two excitatory inputs, the mossy fibers and the climbing fibers. While mossy fibers provide the input for generating the motor program of the cerebellum by activating granule cells, climbing fibers provide the error signals that adapt and optimize the motor program by modulating the activity of Purkinje cells, the only output neurons of the cerebellum [12].

In the last years evidence from anatomical, neuroimaging, clinical, and behavioral studies, indicated that the cerebellum is engaged not only in motor control but also in cognitive and affective functions [13]. By controlling the movement of all muscles of the body, the cerebellum also generates our emotional face expression and is involved in our verbal communication. It also executes all mental processes that lead to behavior and contributes to cognition. Adequate cognition can be defined by a rapid and adequate reaction to our surroundings!

## Function of α6GABA_A_Rs in the Cerebellum

α6GABA_A_Rs are widely distributed in cerebellar granule cells and are highly concentrated at the Golgi cell-granule cell synapses [14]. Due to their specific localization, high GABA sensitivity and long open probability, they are of central importance for the function of the cerebellum. The inhibitory Golgi cells and the excitatory granule cells are simultaneously activated by mossy fibers. Due to the time required until GABA is then released at the Golgi cell-granule cell synapses, α6GABA_A_Rs are activated only after the first action potentials have been elicited in granule cells. Due to their high concentration at and around the Golgi cell-granule cell synapses, they then reduce the intensity and duration of granule cell responses without significantly changing the precision of the first spike [15].

α6GABA_A_Rs thus shorten, better define and separate individual pieces of information from the complex sensory-motor input of the cerebellum that rapidly has to elicit subsequent and distinct activities of the same muscle groups. By that, they mediate a correctly timed and precise coordination of all muscle groups that execute our behavior. Inadequate function of α6GABA_A_Rs results in badly separated and partially overlapping individual inputs to different muscle groups and in simultaneous activation of antagonistic muscles, thus slowing down movements and reactions.

But the excitability of granule cells can be additionally regulated by GABA tonically released from glial cells and acting on α6GABA_A_Rs located at cell bodies, axons, and parallel fibers of granule cells [14,16]. This limits the total amount of information entering the cerebellum, thus reducing information overflow and allowing to concentrate on important and urgent tasks that are characterized by a strong (high frequency) cerebellar input. Information overflow is thought to play a role in stress disorders, anxiety disorders, schizophrenia, attention deficit hyperactivity disorder, and many other psychiatric disorders [6].

## Different Parts of the Cerebellum Contribute to Motor- and Higher-Brain-Functions

Physiological and anatomical investigations have demonstrated that sensory-motor afferents from the spinal cord and cerebral cortex are connected to cerebellar microcircuitries located in the anterior part of the cerebellum, only. Microcircuitries in the posterior part of the cerebellum obtain information from other parts of the brain.

Granule cell activity thus not only represents motor and sensory context, but also nonmotor aspects of contextual information such as expectation of reward, future movement planning, and contextual emotional or cognitive information [13]. fMRI studies and anatomical investigations supported these considerations by demonstrating that different parts of the cerebellum are activated during movement, language, working memory, or social and emotional task processing. Due to the similar microcircuitries throughout the cerebellum, it can be assumed that the respective parts of the posterior cerebellum also collect multiple pieces of contextual information from different brain regions involved in the respective task. α6GABA_A_Rs in these microcircuitries thus probably also regulate the amount and quality of information entering the posterior cerebellum, resulting in final decisions on appropriate actions. In any case, it was concluded that the cerebellum modulates thought, cognition, and emotion, in the same way that it modulates motor control [13].

The cerebellar output via Purkinje cells and deep cerebellar nuclei (DCN) neurons not only is connected to motor areas that mediate the activity of all muscles of the body but also to large parts of the brain via “closed-loop circuits” [17]. These connections provide feedback on the activity of the cerebellum to all brain regions contributing to its activity. Such feedback is essential for the judgement and future improvement of cerebellar activities and thus, for learning by doing, and memory. A suboptimal function of the cerebellum and of their α6GABA_A_Rs thus not only leads to cognitive and behavioral disturbances, but also to their solidification via the information stored in the involved brain regions and circuits. On suboptimal function of the cerebellum, the subjective reality perceived by the brain thus differs from objective reality. This discrepancy might be partially responsible for the psychiatric problems of the disorders. Selective enhancement of α6GABA_A_R activity can thus also correct cognitive and behavioral disturbances!

In agreement with its different functions, lesions of the anterior lobe of the cerebellum causes the cerebellar motor syndrome, whereas lesions in the “limbic cerebellum (vermis and fastigial nucleus) cause a “cerebellar cognitive affective syndrome” [18], that involves disorders of attentional control, emotional control, and social skills as seen in patients with autism spectrum [19] and psychosis spectrum disorders [20]. This clearly demonstrates that symptoms of all these disorders can be elicited by malfunctions of the cerebellum.

## Impaired Structure and/or Function of the Cerebellum in Multiple Neuropsychiatric Disorders

*In vivo* fMRI-investigations, behavioral studies, and post-mortem analyses demonstrated an impaired structure and/or function of the cerebellum of patients suffering from Angelman syndrome, Down syndrome, essential tremor, tic disorders, attention deficit-hyperactivity disorder, obsessive-compulsive disorder, Huntington disease, schizophrenia, autism spectrum disorder, stress-associated disorder, anxiety disorder, depression/bipolar disorder, or alcohol use disorder [6]. An impaired structure and/or function of the cerebellum was also observed in animal models of the respective disorders using behavioral, *in vivo* electrophysiological, and post mortem analyses. This implies a variable imbalance in the expression and/or function of α6GABA_A_Rs in these disorders. Depending on the exact nature and location of this imbalance, different brain regions will be influenced via closed-loop circuit connections of the cerebellum, thus contributing to the different symptoms of the individual disorders.

## Changes in α6GABA_A_R Expression or Function Associated with Symptoms of Neuropsychiatric Disorders

Such changes in the expression or function of α6GABA_A_Rs actually have been demonstrated in multiple human studies and animal models investigating neuropsychiatric disorders. For instance, a variety of genetic studies indicate an association of *GABRA6* variants and neuropsychiatric phenotypes, such as epilepsy, stress disorder, anxiety disorder, major depression, panic disorder, bipolar disorder, schizophrenia, or alcohol-associated disorder [6]. Most if not all of the *GABRA6* variants so far investigated in these studies, cause a changed expression and/or altered function of the *GABRA6* gene. In addition, post-mortem changes in the expression of α6 subunits have also been demonstrated in schizophrenia, mood disorders, autism, and alcohol use disorders [6].

Similar changes in the expression of α6GABA_A_Rs have also been demonstrated in animal models of neuropsychiatric disorders. Interestingly, the α6 subunit changes in the cerebellum of subjects with schizophrenia were similar to those observed in rats chronically treated with the N-methyl-D-aspartate (NMDA) channel blocker PCP, which elicits schizophrenia-like symptoms in humans as well as in animal models [21].

Stress is a major vulnerability factor for neuropsychiatric disorders. In rodents, stress can modulate miRNA expression in pregnant females, leading to altered transcriptomic brain profiles in their offspring [22,23]. Similar miRNA alterations are also observed in human psychiatric disorders. miRNAs can also reduce the expression of GABA_A_R α6 subunits [24,25]. In adolescent rats, chronic maternal separation stress caused a reduced α6 subunit expression in hippocampus and elicited a depressive behavior [26]. In humans, stress during pregnancy, such as family discord, or divorce, or in early life, such as disturbances of the infant-parent relationship, social separation, child neglect, or abuse, also can negatively influence brain development of the offspring and increase the risk of behavioral, emotional, and cognitive problems and later onset-disorders, such as ADHD, autism, and schizophrenia [27–29]. Emerging evidence from animal and human studies suggests that the cerebellum also plays a role in stress responses [30], in fear and anxiety-related disorders [31], autism spectrum disorder, schizophrenia, addiction [32], and alcohol use disorders [33]. All these long-lasting pathologic consequences of prenatal and antenatal stress can be enhanced or reduced by genetic predisposition. A changed expression of α6GABA_A_Rs could thus influence the susceptibility for these disorders [6].

## α6GABA_A_Rs Outside of the Cerebellum might Contribute to Effects of α6-modulators

α6GABA_A_Rs, however, are not only expressed in granule cells of the cerebellum, but also in other parts of the nervous system and in peripheral tissues [6]. Part of the actions of α6-modulators in reducing the symptoms of tic-disorders [8,9], schizophrenia [10], and essential tremor [11] in the respective animal models, might thus also have been generated outside the cerebellum. Purkinje cells provide the output of cerebellar activity to DCN neurons as a continuous chain of action potentials with alternating frequency, that carry the information for the activity and timing of individual muscle groups. Since strong signals elicit longer action potential sequences than weak signals, decoding and separation of the individual pieces of information is again necessary to adequately direct muscle activity, and might also be necessary for modulating thought, emotion, and social behavior [13]. α6GABA_A_Rs expressed at low abundance in the brain regions involved, might contribute to this process, and might partially mediate the effects of α6-modulators in the disorders investigated so far.

## α6GABA_A_Rs in Trigeminal Ganglia Can Modulate Trigeminal-Related Pain

A mechanism partially similar to that in the cerebellum has been identified in trigeminal ganglia, where α6GABA_A_Rs are expressed in >74% of all neurons as well as in satellite glia cells [34,35]. Although the cell bodies of mammalian sensory ganglia are devoid of synaptic contacts, GABA can induce chloride currents in all trigeminal neurons examined [34]. It was thus hypothesized that frequently firing sensory neurons can lead to elevated extracellular K^+^ concentrations during repolarization of neurons between action potentials, which might induce GABA release from satellite glia and neurons [34,35]. The high GABA sensitivity and long open probability of α6GABA_A_Rs might thus facilitate an efficient feedback inhibition of firing of trigeminal neurons [6,35]. This mechanism presumably is similar to that eliciting tonic inhibition of granule cells in the cerebellum, reducing information overflow.

Involvement of α6GABA_A_Rs in trigeminal-related pain perception was supported by the finding that a 30% reduction of α6 subunits in the trigeminal ganglia caused hyperalgesia to temporomandibular joint inflammation [36]. α6-modulators should thus enhance this weak tonic inhibition on trigeminal neurons and reduce trigeminal-related pain. This was demonstrated in animal models of trigeminal neuropathic pain [37], as well as those of migraine-like pain, where α6-modulators abolished and prevented pathological changes associated with migraine as well as migraine-like pain symptoms in a manner mimicked by two standard migraine medications with different mechanisms of action [35,38].

## Summary

In summary, the extensive ability of α6-modulators for reducing symptoms of multiple neurologic and psychiatric disorders, is well supported by the specific localization, electrophysiological properties, and function of α6GABA_A_Rs in the cerebellum and other parts of the nervous system. The cerebellum not only exhibits a central role in movement coordination but is also involved in all our physical and emotional behavior, and cognitive functions. Lesions of the posterior cerebellum of humans can elicit a cerebellar cognitive affective syndrome, and patients suffering from a variety of neurologic and psychiatric disorders exhibit structural and/or functional changes in the cerebellum, that also involve changes in the expression or function of α6GABA_A_Rs [6]. A possible involvement of α6GABA_A_Rs in these disorders is also supported by genetic studies investigating *GABRA6* variants in epilepsy, stress, anxiety, panic, and mood disorders, as well as by post mortem changes of α6GABA_A_R expression in these disorders [6].

α6GABA_A_Rs are of central importance for an adequate function of the cerebellum. They enhance the precision of sensory inputs, enable rapid and coordinated movement and adequate responses to our environment, and strongly influence cognition, emotion, and social behavior. Tonic inhibition mediated by α6GABA_A_Rs regulates the amount of information entering the cerebellum and protects the cerebellum from information overflow. Information overflow is thought to play a role not only in many psychiatric disorders, but also in stress situations, when multiple sensory informations, considerations, and emotions, pointing in different directions, are coming together, thus impeding a rapid and optimal response. This might be even more of a problem in disorders with suboptimal functioning of α6GABA_A_Rs [6]. Enhancing the function of α6GABA_A_Rs might improve the integration of sensory and emotional input and increase the threshold for incoming information. This conclusion is supported by recent findings that a granule cell-specific deletion of GABA_A_R δ subunits in the cerebellum of mice resulted in a differential activation of many cortical and subcortical brain areas involved in cognition, anxiety-like and stress related behaviors in females [39]. If stress can be elicited by a reduced tonic inhibition of granule cells, α6-modulators that also modulate α6βδ GABA_A_Rs [6], should alleviate stress symptoms!

## Perspective

So far, the effectiveness of α6-modulators has only been investigated in animal models of human disorders. In preclinical studies, α6-modulators exhibit no sedation, no movement disturbances, or other benzodiazepine-like side effects, a good metabolic stability on human liver microsomes, good pharmacokinetics in plasma and brain, excellent oral bioavailability, and no liver, kidney, or other signs of toxicity [37,38,40]. Future human studies must investigate their safety and possible side effects, and clarify to which extent individual symptoms of the respective disorders can be reduced by α6-modulators in patients during acute and chronic dosing.

α6-modulators cannot directly activate α6GABA_A_Rs but only enhance the action of GABA at those α6GABA_A_Rs required for a task. They thus exhibit much less side effects than compounds directly activating all α6GABA_A_Rs. If α6GABA_A_Rs are functioning optimally by enhancing the precision of cerebellar input and protecting the brain from information overflow, their function cannot be improved by α6-modulators. Since α6GABA_A_Rs only can be activated after granule cells have started to fire, over-stimulation of these receptors also cannot block the information flow into the cerebellum. This explains why these compounds did not show any side effects in so far 9 different animal studies. Due to their therapeutic potential in multiple neurologic and psychiatric disorders, α6-modulators might open new possibilities for the treatment of a variety of neuropsychiatric disorders and might also help to better cope with stress and its consequences.

## Limitations

So far, the beneficial actions of α6-modulators have been demonstrated in animal models of neuropsychiatric disorders, only. But due to the complexity of psychiatric disturbances in man, efficacy in humans cannot be assumed based on animal data alone. Thus, clinical trials investigating a possible therapeutic use of α6-modulators are required to clarify, whether and to which extent these drugs can ameliorate the symptoms of the individual neuropsychiatric disorders. Nevertheless, the expression of α6-subunits and α6GABA_A_Rs is highly conserved in cerebellar granule cells of teleost fish, chicken, rodents [41], and humans [42], and thus throughout the whole span of vertebrate evolution. In addition, all vertebrates have a cerebellum with essentially the same well-defined circuitry and small number of cell types [41]. Essential brain circuits developed and optimized during evolution are thus not changed in humans, but have been extended to support language, and additional cognitive, emotional, and social task processing. There is thus no reason why an appropriate therapeutic action correcting a dysfunction of an essential brain circuit, should not improve the symptoms of patients! This conclusion is supported by another essential brain circuit, the basal ganglia. Their dopaminergic and additional input from different brain regions, intrinsic organization, and striatal projection neurons expressing dopamine D1- or dopamine D2-receptors, are also highly conserved throughout the vertebrate evolution [43]. A deficit in striatal dopamine in Parkinson´s disease seemed to be causally related to this disorder and suggested the highly successful “dopamine replacement therapy” with L-DOPA [44]. A deficit of GABA-synthesizing enzymes in the cerebellum of schizophrenia patients [42] might also be causally related to this disorder. Why then should not enhancement of the function of α6GABA_A_Rs in the cerebellum improve at least some symptoms of schizophrenia, as demonstrated in respective animal models [10]?

## References

[1] Sieghart W (1995). Structure and pharmacology of γ-aminobutyric acid_A_ receptor subtypes. Pharmacological Reviews.

[2] Olsen RW, Sieghart W (2008). International Union of Pharmacology. LXX. Subtypes of gamma-aminobutyric acid(A) receptors: classification on the basis of subunit composition, pharmacology, and function. Update. Pharmacological Reviews.

[3] Rudolph U, Mohler H (2014). GABA_A_ receptor subtypes: Therapeutic potential in Down syndrome, affective disorders, schizophrenia, and autism. Annu Rev Pharmacol Toxicol.

[4] Skolnick P (2012). Anxioselective anxiolytics: on a quest for the Holy Grail. Trends in Pharmacological Sciences.

[5] Sieghart W, Savic MM (2018). International Union of Basic and Clinical Pharmacology. CVI: GABA_A_ Receptor Subtype- and Function-selective Ligands: Key Issues in Translation to Humans. Pharmacological Reviews.

[6] Sieghart W, Chiou LC, Ernst M, Fabjan J, Savic MM, Lee MT (2022). alpha6-containing GABA_A_ receptors: functional roles and therapeutic potentials. Pharmacological Reviews.

[7] Varagic Z, Ramerstorfer J, Huang S, Rallapalli S, Sarto-Jackson I, Cook J (2013). Subtype selectivity of alpha+beta- site ligands of GABA receptors - identification of the first highly specific positive modulators at alpha6beta2/3gamma2 receptors. Br J Pharmacol.

[8] Chiou LC, Lee HJ, Ernst M, Huang WJ, Chou JF, Chen HL (2018). Cerebellar alpha6-subunit-containing GABA_A_ receptors: a novel therapeutic target for disrupted prepulse inhibition in neuropsychiatric disorders. Br J Pharmacol.

[9] Cadeddu R, Knutson DE, Mosher LJ, Loizou S, Odeh K, Fisher JL, Cook JM, Bortolato M (2021). The α6 GABA_A_ receptor positive allosteric modulator DK-I-56-1 reduces tic-related behaviors in mouse models of Tourette syndrome. Biomolecules.

[10] Lee MT, Mouri A, Kubota H, Lee HJ, Chang MH, Wu CY (2022). Targeting α6GABA_A_ receptors as a novel therapy for schizophrenia: A proof-of-concept preclinical study using various animal models. Biomed Pharmacother.

[11] Huang YH, Lee MT, Hsueh HY, Knutson DE, Cook J, Mihovilovic MD (2023). Cerebellar alpha6GABA_A_ receptors as a therapeutic target for essential tremor: proof-of-concept study with ethanol and pyrazoloquinolinones. Neurotherapeutics.

[12] D’Angelo E, Solinas S, Mapelli J, Gandolfi D, Mapelli L, Prestori F (2013). The cerebellar Golgi cell and spatiotemporal organization of granular layer activity. Frontiers in Neural Circuits.

[13] Schmahmann JD, Guell X, Stoodley CJ, Halko MA (2019). The Theory and Neuroscience of Cerebellar Cognition. Annual Review of Neuroscience.

[14] Nusser Z, Sieghart W, Somogyi P (1998). Segregation of different GABA_A_ receptors to synaptic and extrasynaptic membranes of cerebellar granule cells. J Neurosci.

[15] Nieus TR, Mapelli L, D’Angelo E (2014). Regulation of output spike patterns by phasic inhibition in cerebellar granule cells. Frontiers in Cellular Neuroscience.

[16] Lee S, Yoon BE, Berglund K, Oh SJ, Park H, Shin HS (2010). Channel-mediated tonic GABA release from glia. Science.

[17] Schmahmann JD (1991). An emerging concept. The cerebellar contribution to higher function. Archives of Neurology.

[18] Schmahmann JD, Sherman JC (1998). The cerebellar cognitive affective syndrome. Brain : A Journal of Neurology.

[19] D’Mello AM, Stoodley CJ (2015). Cerebro-cerebellar circuits in autism spectrum disorder. Frontiers in Neuroscience.

[20] Schmahmann JD, Weilburg JB, Sherman JC (2007). The neuropsychiatry of the cerebellum - insights from the clinic. Cerebellum.

[21] Bullock WM, Bolognani F, Botta P, Valenzuela CF, Perrone-Bizzozero NI (2009). Schizophrenia-like GABAergic gene expression deficits in cerebellar Golgi cells from rats chronically exposed to low-dose phencyclidine. Neurochem Int.

[22] Rinaldi A, Vincenti S, De Vito F, Bozzoni I, Oliverio A, Presutti C (2010). Stress induces region specific alterations in microRNAs expression in mice. Behavioural brain research.

[23] Zucchi FC, Yao Y, Ward ID, Ilnytskyy Y, Olson DM, Benzies K (2013). Maternal stress induces epigenetic signatures of psychiatric and neurological diseases in the offspring. PLoS One.

[24] Gong Y, Wu CN, Xu J, Feng G, Xing QH, Fu W (2013). Polymorphisms in microRNA target sites influence susceptibility to schizophrenia by altering the binding of miRNAs to their targets. Eur Neuropsychopharmacol.

[25] Gonda X, Sarginson J, Eszlari N, Petschner P, Toth ZG, Baksa D (2017). A new stress sensor and risk factor for suicide: the T allele of the functional genetic variant in the *GABRA6* gene. Scientific Reports.

[26] Yang L, Xu T, Zhang K, Wei Z, Li X, Huang M (2016). The essential role of hippocampal alpha6 subunit-containing GABA_A_ receptors in maternal separation stress-induced adolescent depressive behaviors. Behavioural Brain Research.

[27] Ronald A, Pennell CE, Whitehouse AJ (2010). Prenatal Maternal Stress Associated with ADHD and Autistic Traits in early Childhood. Frontiers in Psychology.

[28] Gunn BG, Cunningham L, Mitchell SG, Swinny JD, Lambert JJ, Belelli D (2015). GABA_A_ receptor-acting neurosteroids: a role in the development and regulation of the stress response. Frontiers in Neuroendocrinology.

[29] Beversdorf DQ, Stevens HE, Margolis KG, Van de Water J (2019). Prenatal Stress and Maternal Immune Dysregulation in Autism Spectrum Disorders: Potential Points for Intervention. Curr Pharm Des.

[30] Moreno-Rius J (2019). The cerebellum under stress. Frontiers in Neuroendocrinology.

[31] Moreno-Rius J (2018). The cerebellum in fear and anxiety-related disorders. Progress in Neuro-Psychopharmacol and Biol Psychiatry.

[32] Carta I, Chen CH, Schott AL, Dorizan S, Khodakhah K (2019). Cerebellar modulation of the reward circuitry and social behavior. Science.

[33] Rompala GR, Simons A, Kihle B, Homanics GE (2018). Paternal Preconception Chronic Variable Stress Confers Attenuated Ethanol Drinking Behavior Selectively to Male Offspring in a Pre-Stress Environment Dependent Manner. Frontiers in Behavioral Neuroscience.

[34] Hayasaki H, Sohma Y, Kanbara K, Maemura K, Kubota T, Watanabe M (2006). A local GABAergic system within rat trigeminal ganglion cells. Eur J Neurosci.

[35] Fan PC, Lai TH, Hor CC, Lee MT, Huang P, Sieghart W (2018). The alpha6 subunit-containing GABA_A_ receptor: A novel drug target for inhibition of trigeminal activation. Neuropharmacology.

[36] Puri J, Vinothini P, Reuben J, Bellinger LL, Ailing L, Peng YB (2012). Reduced GABA(A) receptor alpha6 expression in the trigeminal ganglion alters inflammatory TMJ hypersensitivity. Neuroscience.

[37] Vasovic D, Divovic B, Treven M, Knutson DE, Steudle F, Scholze P (2019). Trigeminal neuropathic pain development and maintenance in rats are suppressed by a positive modulator of alpha6 GABA_A_ receptors. European journal of pain.

[38] Tzeng HR, Lee MT, Fan PC, Knutson DE, Lai TH, Sieghart W (2021). alpha6GABA_A_ Receptor Positive Modulators Alleviate Migraine-like Grimaces in Mice via Compensating GABAergic Deficits in Trigeminal Ganglia. Neurotherapeutics.

[39] Rudolph S, Guo C, Pashkovski SL, Osorno T, Gillis WF, Krauss JM (2020). Cerebellum-Specific Deletion of the GABA_A_ Receptor delta Subunit Leads to Sex-Specific Disruption of Behavior. Cell Rep.

[40] Knutson DE, Kodali R, Divovic B, Treven M, Stephen MR, Zahn NM (2018). Design and Synthesis of Novel Deuterated Ligands Functionally Selective for the gamma-Aminobutyric Acid Type A Receptor (GABA_A_R) alpha6 Subtype with Improved Metabolic Stability and Enhanced Bioavailability. J Med Chem.

[41] Bahn S, Harvey RJ, Darlison MG, Wisden W (1996). Conservation of gamma-aminobutyric acid type A receptor alpha 6 subunit gene expression in cerebellar granule cells. Journal of Neurochemistry.

[42] Bullock WM, Cardon K, Bustillo J, Roberts RC, Perrone-Bizzozero NI (2008). Altered expression of genes involved in GABAergic transmission and neuromodulation of granule cell activity in the cerebellum of schizophrenia patients. The American Journal of Psychiatry.

[43] Grillner S, Robertson B (2016). The Basal Ganglia Over 500 Million Years. Current Biology.

[44] Hornykiewicz O (2017). L-DOPA. Journal of Parkinson’s Disease.

